# Supplementary material for: ExplaiNN: interpretable and transparent neural networks for genomics
Source: Genome Biol. 2023 Jun 27;24:154. doi: 10.1186/s13059-023-02985-y (PMC10303849; doi:10.1186/s13059-023-02985-y)
Supplement: Supplementary file 2 — Additional file 2. Supplementary figures. [file 13059_2023_2985_MOESM2_ESM.docx]

**
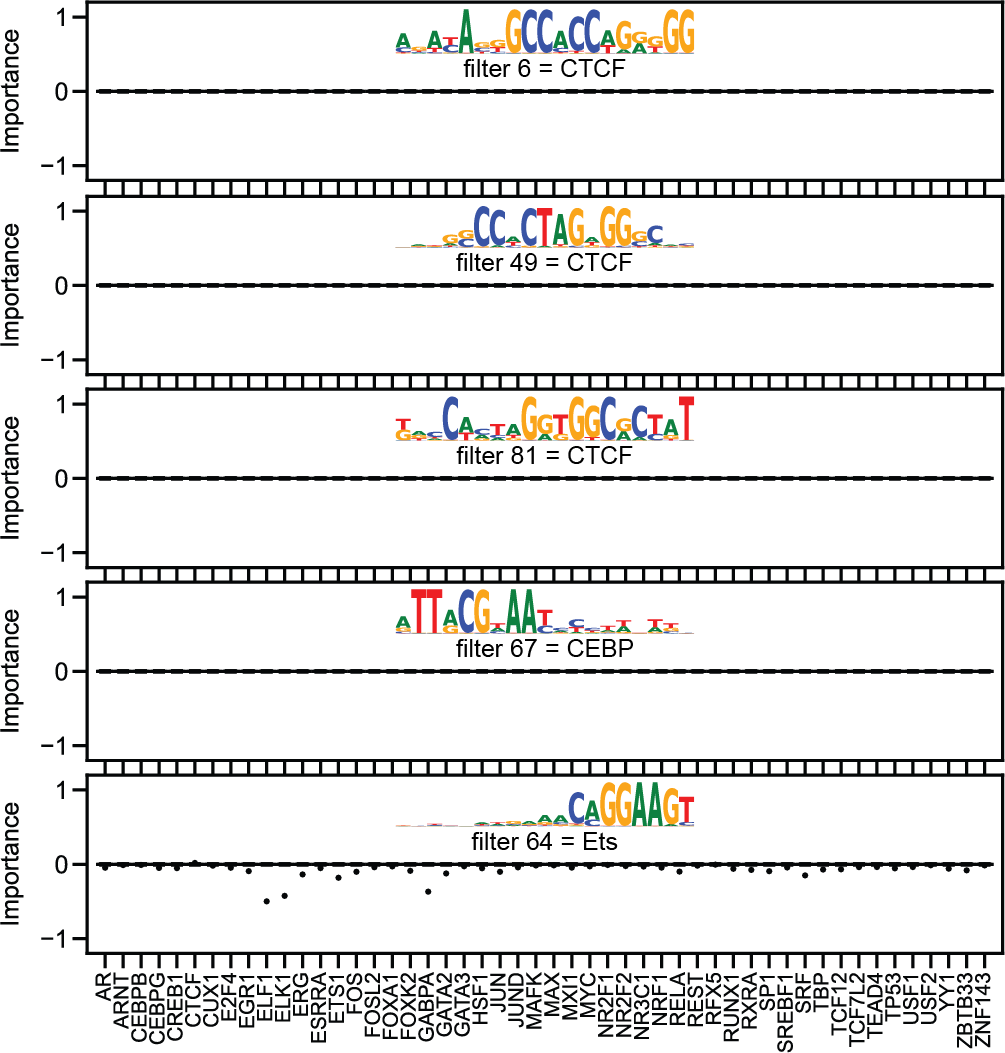
**

**Fig. S1** From top to bottom, visualization of importance scores for three units annotated as CTCF, one unit annotated as CEBP, and one unit annotated as Ets from an ExplaiNN model trained using 100 units on predicting the binding of 50 TFs in OCRs. OCR, open chromatin region


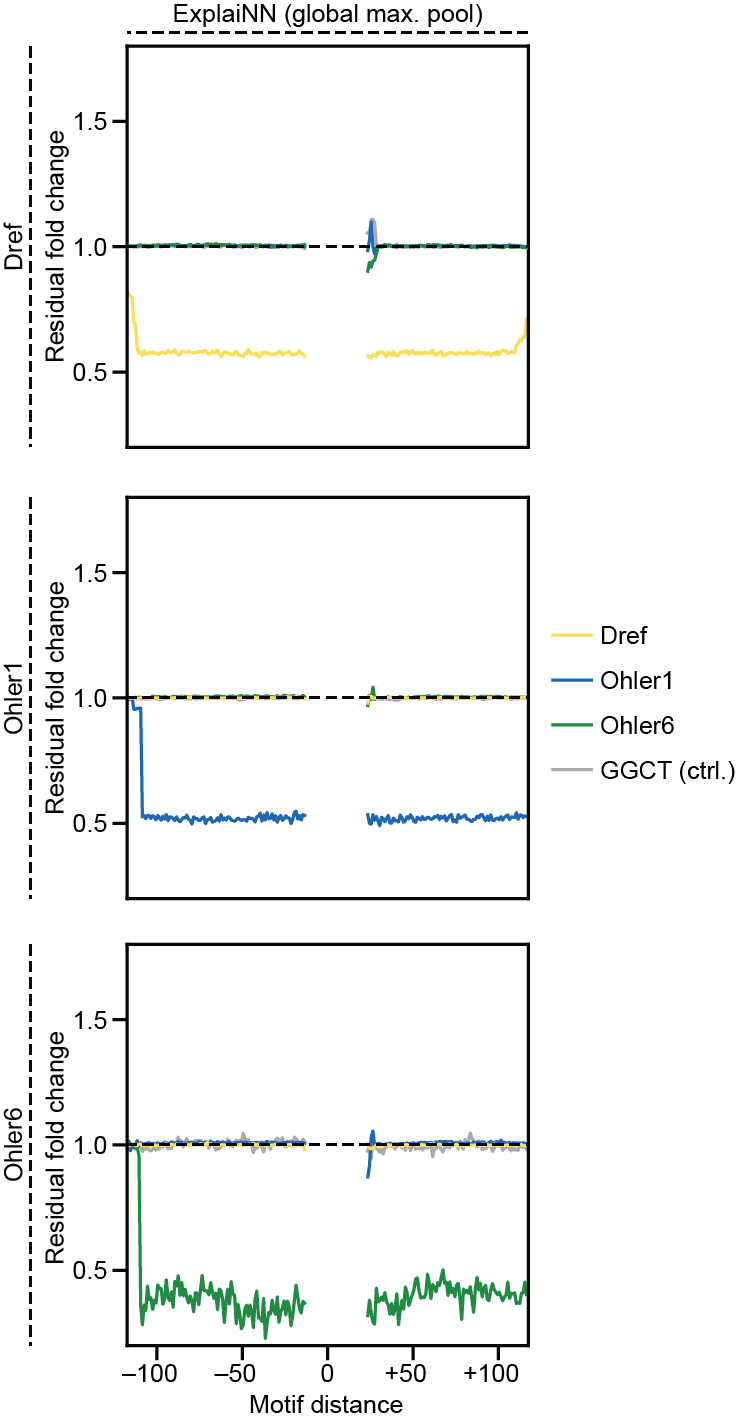


**Fig. S2** Cooperativity (residual fold change; *y*-axis) plotted as a function of distance (*x*-axis) between the motifs of the housekeeping TFs Dref (top row, yellow), Ohler1 (middle row; blue), and Ohler6 (bottom row; green) for an ExplaiNN model in which the fully connected layers of each unit had been replaced with a global max pooling layer. The 5-mer GGGCT is provided as a negative control (light gray). TF, transcription factor


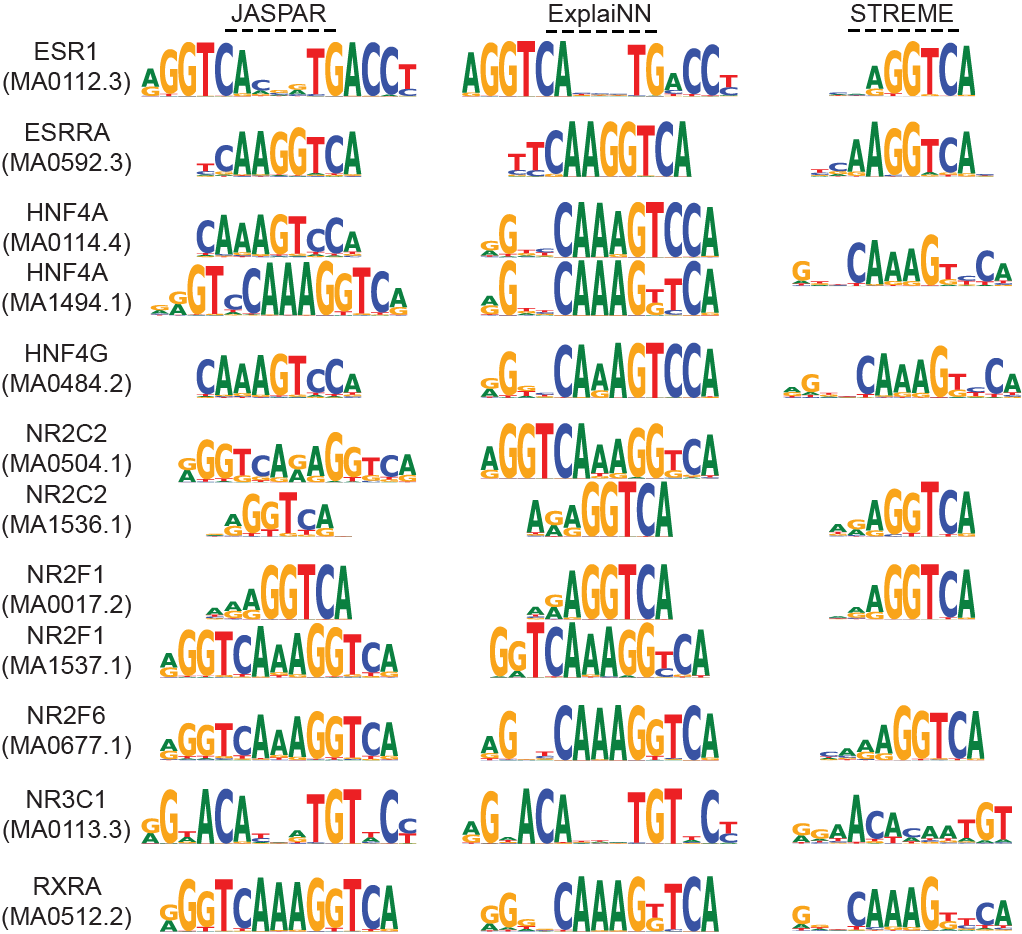


**Fig. S3** Logos derived using ExplaiNN or STREME [34] for the nuclear receptors ESR1, ESRRA, HNF4A, HNF4G, NR2C2, NR2F1, NR2F6, NR3C1, and RXRA on in vivo datasets describing the binding of these TFs to OCRs. For comparison, the JASPAR [18] logos for these TF profiles are shown: MA0112.3 (ESR1), MA0592.3 (ESRRA), MA0114.4 and MA1494.1 (HNF4A), MA0484.2 (HNF4G), MA0504.1 and MA1536.1 (NR2C2), MA0017.2 and MA1537.1 (NR2F1), MA0677.1 (NR2F6), MA0113.3 (NR3C1), and MA0512.2 (RXRA).


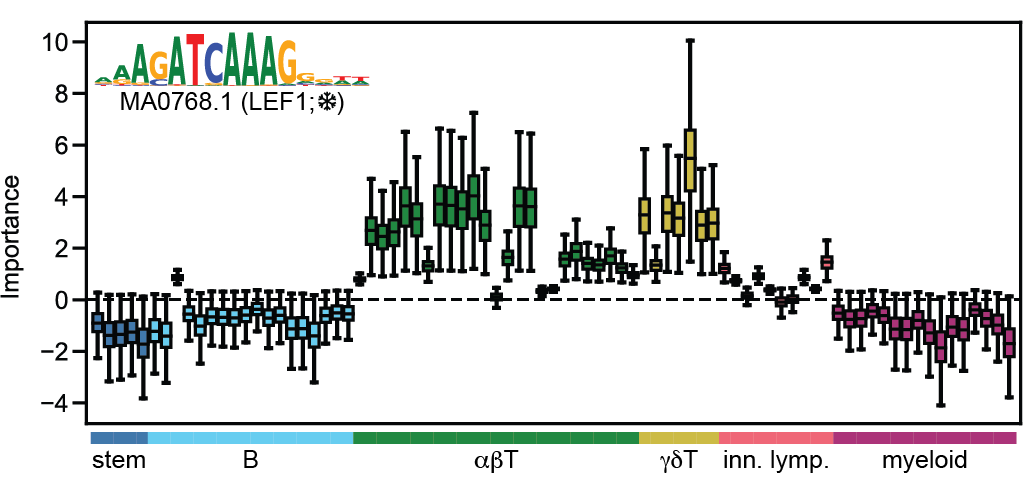


**Fig. S4** Visualization of importance scores coloured by lineage of a unit from an ExplaiNN model initialized with 300 JASPAR profiles and trained on the AI-TAC dataset [9] with freezing. The unit, which corresponded to the JASPAR profile of Lef1 (MA0768.2), was important for predicting accessibility in T cells, in agreement with the role of this TF in establishing T cell identity [50]. TF, transcription factor


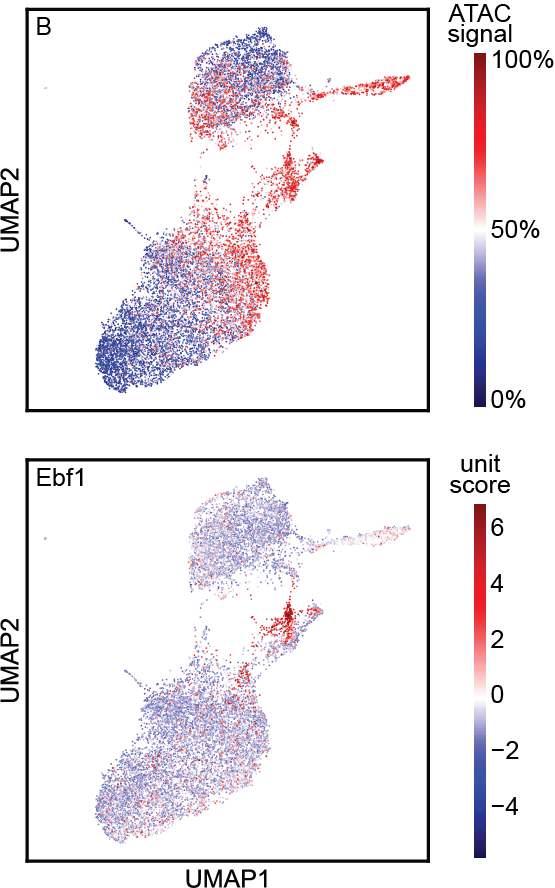


**Fig. S5** ExplaiNN model with 350 units trained on the AI-TAC dataset [9] in which the units had been replaced with 350 different pretrained DanQ [7] models, each predicting the binding of a single TF to the mouse genome. During the training process of the ExplaiNN model, the DanQ models were frozen (i.e., their weights were not modified). TF, transcription factor
